# HDAC3 in action: Expanding roles in inflammation and inflammatory diseases

**DOI:** 10.1111/cpr.13731

**Published:** 2024-08-14

**Authors:** Ruyuan He, Zhuokun He, Tianyu Zhang, Bohao Liu, Minglang Gao, Ning Li, Qing Geng

**Affiliations:** ^1^ Department of Thoracic Surgery Renmin Hospital of Wuhan University Wuhan China; ^2^ Department of Thoracic Surgery Jilin University Changchun China

## Abstract

Inflammation serves as the foundation for numerous physiological and pathological processes, driving the onset and progression of various diseases. Histone deacetylase 3 (HDAC3), an essential chromatin‐modifying protein within the histone deacetylase superfamily, exerts its transcriptional inhibitory role through enzymatic histone modification to uphold normal physiological function, growth, and development of the body. With both enzymatic and non‐enzymatic activities, HDAC3 plays a pivotal role in regulating diverse transcription factors associated with inflammatory responses and related diseases. This review examines the involvement of HDAC3 in inflammatory responses while exploring its therapeutic potential as a target for treating inflammatory diseases, thereby offering valuable insights for clinical applications.

## Graphical abstract

HDAC3, a member of the histone deacetylase (HDAC) superfamily, is involved in the regulation of acetylation/deacetylation processes associated with inflammation‐related diseases. This article reviews the key role of HDAC3 in the process of inflammation, the potential molecular mechanism and the clinical significance of targeted therapy.

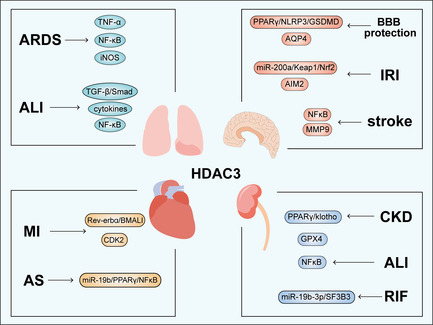

## 1 INTRODUCTION

Inflammation is defined as the pathophysiological response of cells and tissues to pathogenic microbial infection, trauma, metabolic response, etc.^1^, ^2^ It is an immune response by the body to eliminate harmful stimuli and facilitate tissue repair.^1^, ^3^ Vascular endothelial permeability is increased, allowing the release of inflammatory factors and mediators into the circulation to activate inflammatory pathways.^2^ Normally, the protective inflammatory response is transient and controllable^4^; however, when it becomes too intense to be eliminated in a short term period it can have detrimental effects on multiple organs and systems, leading to disease development.^1^, ^2^ Hence, due to their contribution towards numerous acute and chronic diseases and cancers, the regulatory mechanisms of inflammatory pathways have been a long‐standing and worthwhile topic of debate.^3^, ^5^

Epigenetic modifications play a crucial role in regulating gene expression through the reversible regulation of acetylation levels on both histones and non‐histones by enzymes such as histone deacetylases (HDACs) and histone acetyltransferases (HATs).^6^ HDAC3 is a member of the HDAC superfamily in mammalian genomes^7^ and is found within a distinct complex that includes nuclear receptor corepressors NCOR1/NCOR2.^8^ It interacts with various protein chaperones to regulate biological processes extensively studied in recent years, particularly its role in acetylation/deacetylation processes associated with inflammation‐related disease progression.^9^, ^10^ In this review, we present a comprehensive overview of the current understanding regarding the pivotal role of HDAC3 in inflammatory processes, with a specific focus on elucidating the underlying molecular mechanisms and their associated clinical diseases.

## 2 STRUCTURE AND FUNCTION OF HDAC3

### 2.1 The unique structure of HDAC3

HDACs are categorized into distinct classes based on sequence conservation, and they perform specific functions in both the nucleus and cell membrane.^11^ The enzymatic activity of Class I, II, and IV HDACs is dependent on the presence of zinc ions,^12^ whereas Class III HDACs necessitate nicotinamide adenine dinucleotide (NAD) as a cofactor.^13^ Class I HDACs include HDAC1, HDAC2, HDAC3, and HDAC8, which are predominantly localized in the nucleus, whereas HDAC3 exhibits translocation from the nucleus to the cytosol.^11^ Class II HDACs, including HDAC4, HDAC5, HDAC6, HDAC7, HDAC9, and HDAC10, are localized in both the nucleus and cytoplasm and undergo phosphorylation by either protein kinase C or protein kinase D.^14^ Additionally referred to as sirtuins, Class III HDACs enzymes, the NAD‐dependent enzymes, exhibit structural resemblances to the yeast sir2 silencing protein.^15^ The sole member of Class IV, HDAC11, shares sequence identity with the deacetylase domain of both Class I and II HDACs.^16^

The structural similarity of all Class I HDAC enzymes is evident, as they predominantly contain the catalytic core structural domain, C‐terminus, and N‐terminal regions.^17^ HDAC1 and HDAC2 were initially identified in humans; however, unlike HDAC1 and HDAC2, HDAC3 possesses several distinctive structural features.^18^ The local positioning of certain amino acid residues in HDAC3 is distinct, and HDAC3 exhibits distinct structural variations at positions 13 and 29.^19^ The presence of these residue substitutions and differences gives rise to a distinctive molecular structure, which exhibits significant potential for the development of highly selective HDAC3 inhibitors.

### 2.2 The enzymatic activity and non‐enzymatic functions of HDAC3

Among the Class I HDACs, HDAC1 and HDAC2 are present in three inhibitory complexes: NuRD, CoREST, and Sin3A, whereas HDAC3 appears to be selectively recruited to the NCoR/SMRT complex, which interacts with the deacetylase activation domain (DAD) conserved in NCoR or SMRT.^20^ TBL1XR1 and TBL1X, WD40 repeat‐containing proteins, are contained in the NCoR and SMRT complexes to recruit the 19S proteasome and ubiquitination machinery to histones.^21^ Another core component of the complex is the G‐protein pathway repressor 2, however, its role remains unclear.^22^ As a gene switch, the nuclear receptor activates signal‐dependent transcription factors to regulate gene transcription.^23^ Nuclear receptor co‐repressor complexes, comprising HDAC3, associate with ligand‐free nuclear receptors and exert direct repression on gene expression.^24^, ^25^

#### 2.2.1 Enzymatic activity of HDAC3

As previously mentioned, HDAC3 functions as a catalytic component within the stable HDAC3/NCoR/SMRT complex, where its histone deacetylation activity is enhanced by recruitment into the stable co‐repressor complex by repressive transcription factors. The catalytic function of HDAC3 requires DAD –a SANT pattern comprising 57 amino acids in length.^26^ In NCoR and SMRT complexes, this SANT sequence is flanked by a unique 36‐amino acid terminus that facilitates the interaction and activation of NCoR and SMRT with HDAC3.^16^ The crystal structure analysis reveals significant protein–protein interactions between the N‐terminus of HDAC3 and the DAD domain of SMRT.^16^ Notably, HDAC3 exhibits significant instability in its unbound state; however, when sequestered by T‐complex 1, a circular cytoplasmic complex, it undergoes facilitated folding within the cytoplasm,^27^ leading to formation of active enzyme‐containing the NCoR and SMRT complex with ATP dependence.^28^

#### 2.2.2 Non‐enzymatic functions of HDAC3

In addition to its classical enzymatic functions, HDAC3 possesses important and cryptic non‐enzymatic roles.^29^ Specifically, a point mutation in one of the active sites of HDAC3, Y298F, disrupts its deacetylase activity. Partial rescue of hepatic steatosis is observed through point mutations in HDAC3 within the liver of deficient mice, which suppresses the expression of lipogenic genes.^28^ Besides, HDAC3 knockout is lethal and causes embryonic death.^30^ Moreover, dependent on its deacetylase activity, HDAC3 selectively binds to activating transcription factor 3 (ATF3) and inhibits Toll‐like receptor (TLR) signalling; it can also bind ATF2 without NCoR1/SMRT and activate the expression of inflammatory genes.^10^ Currently, the non‐enzymatic function of HDAC3 remains undetermined so the development of drugs targeting HDAC3 still needs to be carefully evaluated.

## 3 HDAC3 REGULATES INFLAMMATION

Inflammation is a vital protective response orchestrated by the body to ensure the elimination of harmful irritants and facilitate tissue healing. Recent studies have elucidated the regulatory role of HDAC3 in inflammatory responses through diverse signalling pathways. However, the regulation of inflammatory response by HDAC3 remains a subject of controversy, contingent upon the specific target and mode of action.

### 3.1 HDAC3 regulate inflammatory mediators and enzymes

**FIGURE 1:**
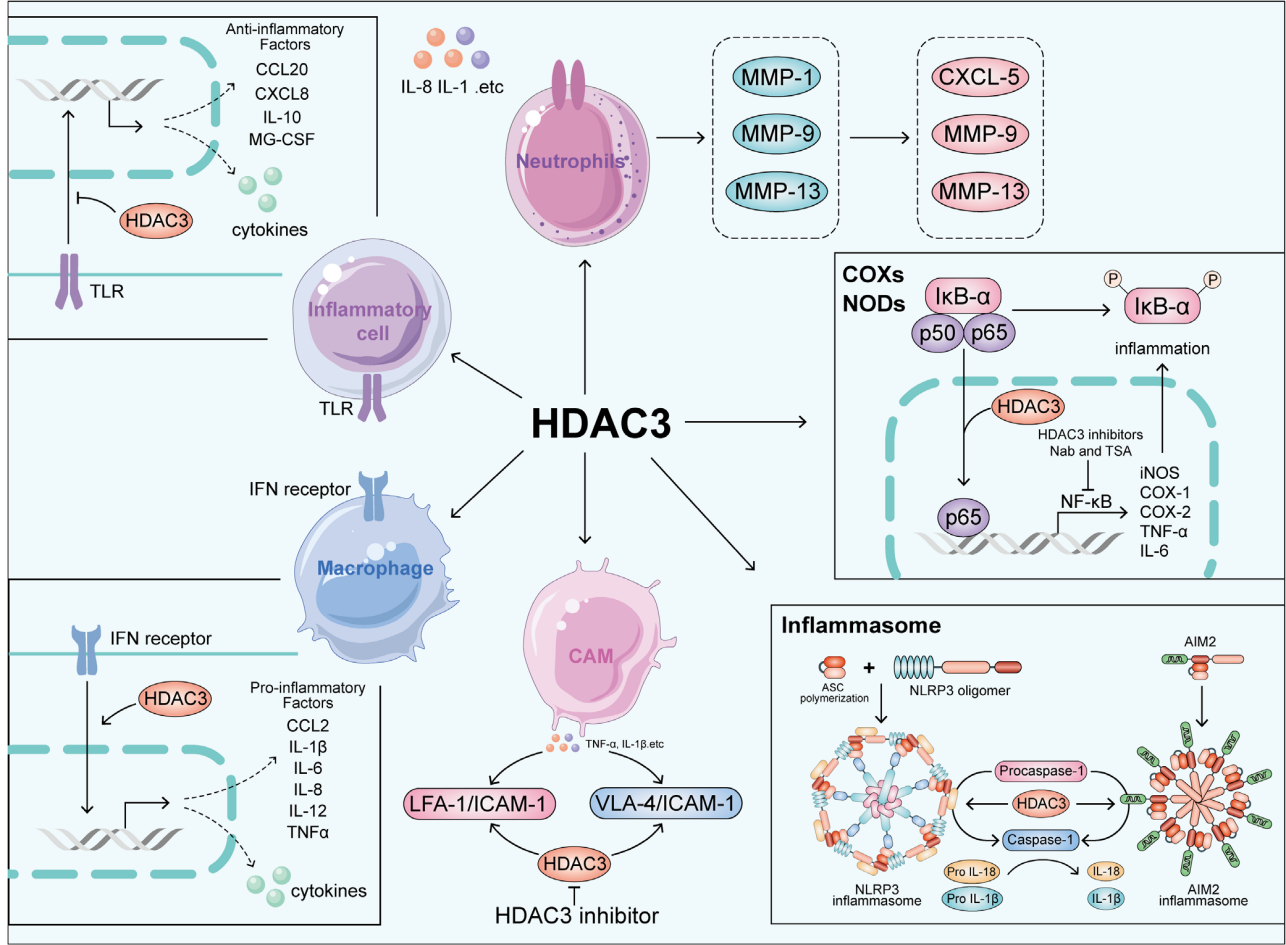
HDAC3 regulates various inflammatory mediators and enzymes, such as cytokines, CAMs, inflammasomes, COXs, NOSs, and MMPs, affects related downstream gene expression, and plays a crucial regulatory role in the inflammatory response.

Inflammatory mediators are induced by endogenous or exogenous factors and subsequently modulate the function of downstream tissues and organs. These mediators primarily induce local inflammatory exudation, allowing plasma proteins and neutrophils, which are normally confined within blood vessels, to extravasate into surrounding tissues via postcapillary venules. Notably, HDAC3 exhibits distinct regulatory relationships with certain inflammatory mediators (Figure 1).

#### 3.1.1 Cytokines

Cytokines represent a diverse group of small regulatory proteins that govern immune cell activity; they include interleukins, interferons, tumour necrosis factor superfamily members, colony‐stimulating factors, chemokines, growth factors, etc.

Class I HDACs predominantly regulate innate immunity‐related inflammatory cytokines through TLR signalling pathways and interferon gene expression modulation.^31^ HDAC3 plays a pivotal role as a crucial epigenetic regulator in the transcriptional control of inflammatory genes. Tumour Necrosis Factor (TNF) serves as a mediator for inflammatory response and immune function regulation.^32^ HDAC3 can suppress TNF expression in monocytes while modulating its post‐transcriptional modification at the transcriptional level. TNF‐α, a key regulator among pro‐inflammatory cytokines within the TNF superfamily,^33^ plays a central role in initiating and sustaining inflammation by coordinating activation and recruitment of inflammatory cells; thus requiring HDAC3 activity for its production and induction.^34^

Given the crucial involvement of HDAC3 in cytokine regulation, its inhibitors hold therapeutic promise across various inflammatory diseases. Sodium butyrate (NaB) is recognized as an effective histone deacetylase inhibitor (HDACI). Studies have supported NaB's potent anti‐inflammatory properties through inhibition of pro‐inflammatory mediators such as TNF‐α, IL‐1β, IL‐6, and IL‐8 along with upregulation of anti‐inflammatory mediator IL‐10.^35^ Both in vitro and in vivo models have demonstrated that HDACIs not only decrease levels of inflammatory cytokines like TNF‐α, IL‐1β, and IL‐6 but also polarized Th1/Th17 cytokines.^36^

#### 3.1.2 Cell adhesion molecules (CAMs)

Cell adhesion molecules (CAMs), such as Vascular cell adhesion molecule‐1 (VCAM‐1) and intercellular cell adhesion molecule‐1 (ICAM‐1), play a pivotal role in orchestrating the inflammatory response.

Trichostatin A (TSA) functions as a potent inhibitor of histone deacetylase, thereby regulating the expression of VCAM‐1 in endothelial cells stimulated by TNF‐α through acetylation.^14^ Furthermore, HDAC3 knockdown in Human Umbilical Vein Endothelial Cells leads to decreased VCAM‐1 expression and subsequent inhibition of monocyte adhesion,^37^ further supporting the involvement of HDAC3 in the regulation of VCAM‐1 expression.

MS‐275, a selective class I HDACI, effectively reduced adhesion molecules such as VCAM‐1 and ICAM‐1, macrophage infiltration, and inflammatory markers including TNF‐α, IL‐1β, and MCP‐1 in animal models.^38^ These findings suggest that the inhibition of HDAC3 can downregulate CAM expression and ultimately lead to an anti‐inflammatory response. However, a study revealed contrasting results.^39^ It was found that ITSA‐1 (an HDAC activator) could activate HDAC activity and reduce VCAM‐1 expression in venous endothelial cells. Conversely, under arterial laminar shear stress in hemodynamics conditions, the expression of HDAC3 decreased, whereas the expression of VCAM‐1 increased, leading to inflammation induction and cell loss. Additionally, another study indicated that overexpression of HDAC3 prevented hyaluronic acid‐induced expression of ICAM‐1 and VCAM‐1.^40^ These observations imply a complex relationship between HDAC3 activity and VCAM‐1 regulation which remains unexplained. However, there is a limited body of research on the regulatory role of HDAC3 in CAMs, with CAMs primarily being utilized as a detection indicator rather than the primary focus of investigation. Therefore, further studies are required to elucidate the specific mechanisms involved while considering HDAC3 and CAMs expression level changes.

#### 3.1.3 Inflammasome

Inflammasomes serve as receptors/sensors of the innate immune system, initiating inflammation in response to exogenous pathogens or endogenous danger signals.

NOD‐like receptor protein 3 (NLRP3) is a crucial member of the NOD‐like receptor family, playing a pivotal role in mediating inflammation. Activation of NLRP3 leads to the cleavage of procaspase‐1 into its active caspase‐1 form, resulting in the maturation and secretion of IL‐1β and IL‐18. The upregulation of histone acetylation at the NLRP3 promoter contributes to the activation of NLRP3 inflammasomes in vascular smooth muscle cells.^41^ Recent studies have demonstrated that HDAC3 facilitates NLRP3‐dependent caspase‐1 activation in vivo, thereby promoting lipopolysaccharide‐induced acute inflammation and high‐fat diet‐induced chronic inflammation.^42^

Besides NLRP3 inflammasomes, HDAC3 is also involved in the regulation of other inflammasomes. Through the induction of cytoplasmic DNA or pathogenic factors such as LPS, IFN‐γ, and IFN‐β, AIM2 recruits apoptosis‐associated speck‐like protein (ASC) and caspase‐1 to facilitate the maturation of IL‐1β, IL‐18, and TNF‐α. This multiprotein complex is also referred to as AIM2 inflammasomes. In vivo and in vitro studies have demonstrated that HDAC3 can modulate AIM2 inflammasome activity.^43^ Furthermore, the utilization of HDAC3 inhibitors has demonstrated their potential in mitigating inflammation through the downregulation of AIM2 inflammasomes.^44^, ^45^

#### 3.1.4 Cyclooxygenases (COXs)

Cyclooxygenases (COXs) are enzymes responsible for the conversion of arachidonic acid to prostaglandins and produce reactive oxygen species as a by‐product.^46^ The interaction between HDAC3 and COXs primarily revolves around the regulation of immune and inflammatory functions. In macrophages deficient in HDAC3, there is a significant increase in constitutive expression of COX‐1. This upregulation may be attributed to hyperactive nuclear receptors binding to an enhancer region upstream of the COX‐1 gene.^47^ Knockdown of HDAC3 partially inhibits endogenous IFN‐β production by increasing COX‐1 expression, thereby impeding the upregulation of IFN‐β response genes.^36^

HDAC3 also regulates COX‐2 expression. MS‐275 has been shown to induce transcription and functional enzyme accumulation of the COX‐2 gene by activating the Nuclear factor kappa‐B (NF‐κB) pathway^48^ or binding to the COX‐2 promoter sequence.^49^ However, NaB and TSA, two HDACIs, were found to block TNF‐α activation synthesized by COX‐2 protein and mRNA, significantly inhibiting COX‐2 activity.^50^ Since post‐transcriptional regulation mainly controls COX‐2 expression, protein acetylation may be required for cytokine activation and stability of COX‐2 mRNA as a plausible explanation; however, this contradiction requires further investigation. However, the regulation of HDAC3 and COX family remains incompletely and systematically investigated. The relationship between changes in expression level and activity of COX proteins with their acetylation state as well as the impact of alterations in protein acetylation on mRNA stability and expression modification are yet to be determined.

#### 3.1.5 Nitric oxide synthases (NOSs)

Nitric oxide synthases (NOSs) encompass a group of enzymes that facilitate the conversion of L‐arginine into nitric oxide (NO), including three NOS isozymes, namely neuronal NOS (nNOS), inducible NOS (iNOS), and endothelial NOS (eNOS). Modulation of NOS activity represents a potential therapeutic strategy for managing inflammatory diseases.

HDAC3 has been identified as a deacetylase for eNOS lysine residues, leading to the inhibition of eNOS activity and counteracting endothelial NO production and vascular function improvement.^51^ Furthermore, HDAC3 can be recruited to the core promoter region of eNOS, inducing histone deacetylation and subsequently inhibiting eNOS transcriptional activity.^52^ HDAC3 inhibits iNOS expression and NO production.^47^, ^53^, ^54^ The latest evidence suggests that the modulation of histone acetylation plays a pivotal role in the activation and expression of iNOS/NO,^55^, ^56^ indicating that targeting histone acetylation modification could be a viable approach for treating inflammatory diseases associated with iNOS activation.

#### 3.1.6 Matrix metalloproteinases (MMPs)

Matrix metalloproteinases (MMPs) are a family of zinc‐dependent endopeptidases that play a crucial role in regulating multiple functions associated with inflammation, including the bioavailability and activity of inflammatory cytokines and chemokines. The expression of MMPs is mediated by acetylation changes catalysed by enzymes belonging to the HDAC family.

MMP9 is primarily derived from neutrophils and has been demonstrated to play a pivotal role in the blood–brain barrier (BBB) disruption.^57^ The protective effect of RGFP966 on the BBB is achieved by modulating the expression levels of HDAC3 in astrocytes and downregulating MMP9.^43^ Additionally, intra‐articular administration of TSA decreased the levels of MMP1, MMP3, and MMP13, thereby attenuating joint injury.^58^ In mouse models of renal transplantation and hepatocellular carcinoma, inhibition of HDAC3 maintains MMPs.^59^, ^60^ These findings confirm the regulatory role of HDAC3 in MMPs regulation and provide a novel perspective for targeted inflammation therapy.

### 3.2 Interactions between HDAC3 and other inflammatory transcription factors

The expression of inflammatory genes is regulated by pro‐inflammatory transcription factors, which are activated in inflammatory diseases and play a pivotal role in amplifying and perpetuating inflammatory processes. HDAC3 exhibits marked selectivity towards distinct transcription factors, thereby influencing their regulation of the inflammatory response.

#### 3.2.1 Nuclear factor erythroid 2‐related factor 2 (Nrf2)

Nuclear factor erythroid 2‐related factor 2 (Nrf2) serves as a pivotal redox‐sensitive regulator of antioxidant protein expression.^61^ By binding to the proximal region of pro‐inflammatory cytokine coding genes, it orchestrates the regulation of numerous antioxidant genes to safeguard cellular integrity and alleviate inflammatory responses.^62^ Previous investigations have demonstrated HDAC3 exerts inhibitory effects on Nrf2 promoter activity.^63^

The efficacy of HDAC3‐targeted Nrf2 in the treatment of inflammation has been validated. Recent evidence suggests that RGFP966 suppresses the activation of the HDAC3/Nrf2 signalling pathway, modulates oxidative stress induced by surgical brain injury.^64^ The activation of the Nrf2/ARE pathway is closely associated with HDACs.^65^ Inhibition of HDAC3 in diabetic mice can delay diabetes‐induced liver injury by activating Nrf2.^66^ Given the significant regulatory impact of HDAC3 on Nrf2, several researchers have proposed using Nrf2 as a biomarker to assess the efficacy of HDACIs.^67^ However, it remains an open question whether upstream factors influencing Nrf2 are also implicated in regulating HDAC3 inhibition and the subsequent alterations in cytoplasmic and nuclear content and activity following the interaction between HDAC3 and Nrf2.

#### 3.2.2 NF‐κB

**FIGURE 2:**
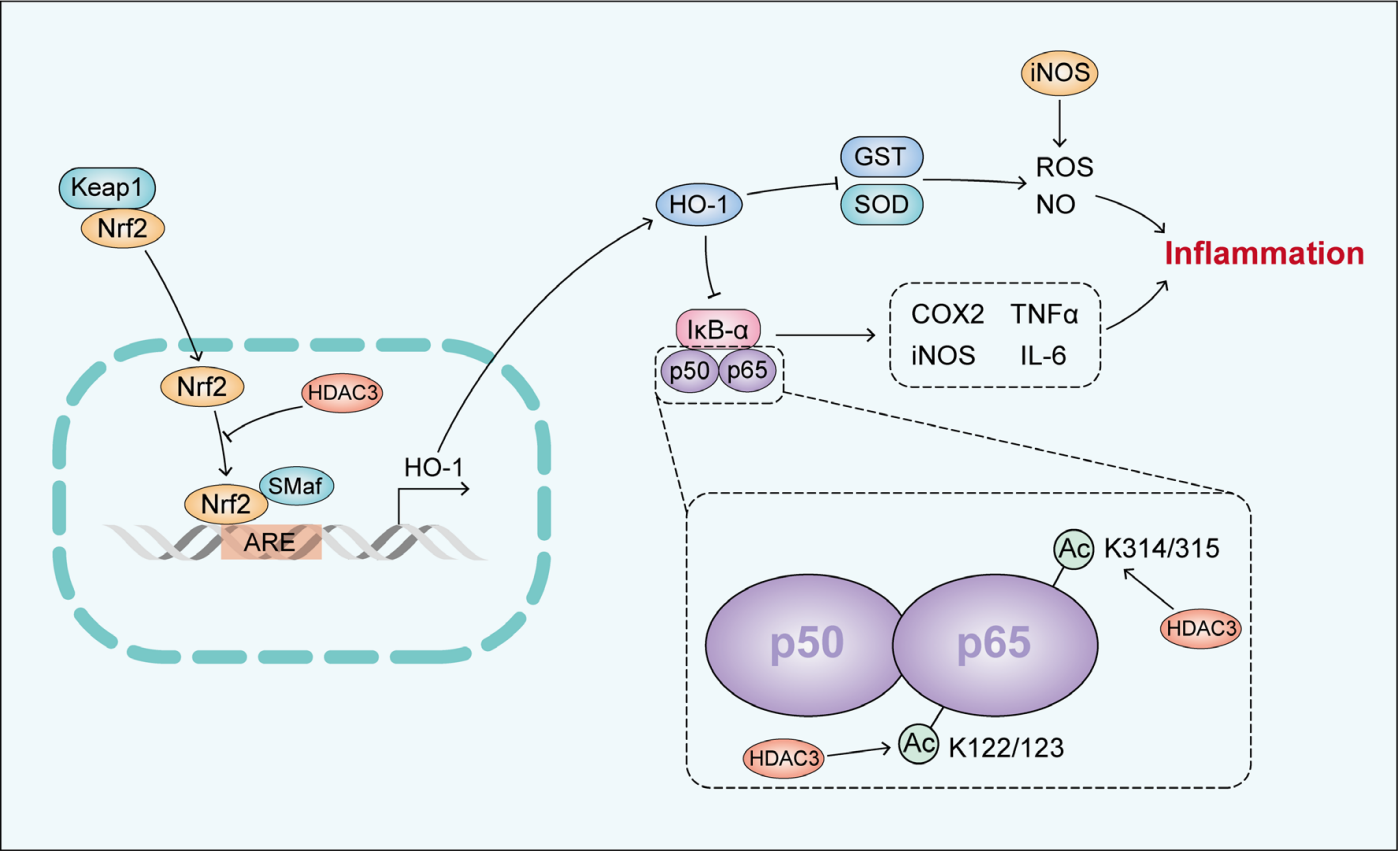
Regulatory role of HDAC3 in Nrf2 and NF‐κB.

The NF‐κB protein complex, consisting of five distinct family members, is crucial for regulating inflammation and immunity.^47^ As a central regulator of inflammatory response,^43^ NF‐κB plays a pivotal role in various inflammatory diseases. The duration and function of nuclear NF‐κB are tightly regulated through reversible acetylation/deacetylation events occurring at distinct sites.^68^ Through deacetylating the p65 subunit and promoting its interaction with the inhibitory subunit iκbα,^69^ HDAC3 indirectly suppresses NF‐κB signalling. NF‐κB can also be regulated by HDAC3 together with Nrf2 (Figure 2). The deletion of HDAC3 may disrupt the balance of NF‐κB‐dependent nuclear transcription and directly result in excessive expression of inflammatory cytokines.^70^

The regulation of NF‐κB by HDAC3 involves multiple other inflammatory mediators and pathways.^70^ Notably, acetylation at different sites has distinct impacts on NF‐κB‐dependent gene expression and NF‐κB transcriptional activity.^71^ HDAC3 positively regulates inflammatory gene expression by modulating lysine 122, 123, 314, 315 deacetylation, whereas deacetylation at lysine 310 exerts an inhibitory effect.^72^

Previous studies have reported that HDACIs modulate inflammation (in vitro or in vivo) by regulating lysine acetylation on NF‐κB p65,^73^, ^74^ thus providing novel insights into the discovery of anti‐inflammatory drugs. However, it is worth noting that due to the intricate regulatory mechanisms governing inflammation, further investigations are required to elucidate acetylation at different sites within the NF‐κB pathway for its activation or inhibition, as well as to explore the specific regulatory mechanisms underlying various HDACIs.

#### 3.2.3 Activating transcription factors (ATFs)

ATFs are a subset of transcription factors belonging to the ATF/CREB family,^75^ which actively participate in inflammation‐associated pathological changes through signal transduction pathways associated with inflammation.^76^ ATF2 is involved in various cellular processes, including cell cycle progression, differentiation, and immune response.^77^ HDAC3 regulates ATF2 transcription by reducing phosphorylation and histone acetylation.^78^ Moreover, HDAC3 selectively suppresses the transcriptional activity of ATF2, thereby participating in the control of pro‐inflammatory cytokine genes such as TNF expression.^79^ HDAC3 can also be recruited and activated by ATF2 during the process of LPS‐induced macrophage activation with its non‐enzymatic activity,^10^ thus facilitating the expression of inflammatory genes. In this context, ATF2 is more inclined towards promoting inflammation through the non‐enzymatic function of HDAC3.^10^ This further emphasizes the significance of the non‐enzymatic activity of HDAC3 in physiological processes.

#### 3.2.4 Peroxisome proliferators‐activated receptors (PPARs)

Peroxisome proliferator‐activated receptors (PPARs) are members of the nuclear receptor superfamily of transcription factors. PPARγ functions as a nuclear transcription factor that plays a crucial role in inflammation and several diseases.^80^, ^81^ In macrophages, the ligand‐binding domain of PPARγ specifically targets the NCoR/HDAC3 complex on promoters of inflammatory genes. HDAC3 deficiency can induce mitochondrial dysfunction associated with impaired PPARγ signal transduction.^82^ HDAC3 inhibition enhances the expression of PPARγ target genes.^83^ This effect may be attributed to the reduction in direct binding between HDAC3 and PPARγ, resulting in increased acetylation of PPARγ and subsequent upregulation of target gene expression.^84^ Recent evidence^85^ indicates that BRD3308, a selective HDAC3 inhibitor, upregulates the expression of PPARγ and suppresses NLRP3‐mediated inflammatory cytokine production, thereby mitigating neuroinflammation. However, the precise regulatory mechanism underlying HDAC3‐mediated inhibition of PPARγ activity remains elusive, necessitating further comprehensive investigation into the associated molecular regulatory pathways.

## 4 THE ROLE OF HDAC3 IN INFLAMMATORY DISEASES

**FIGURE 3:**
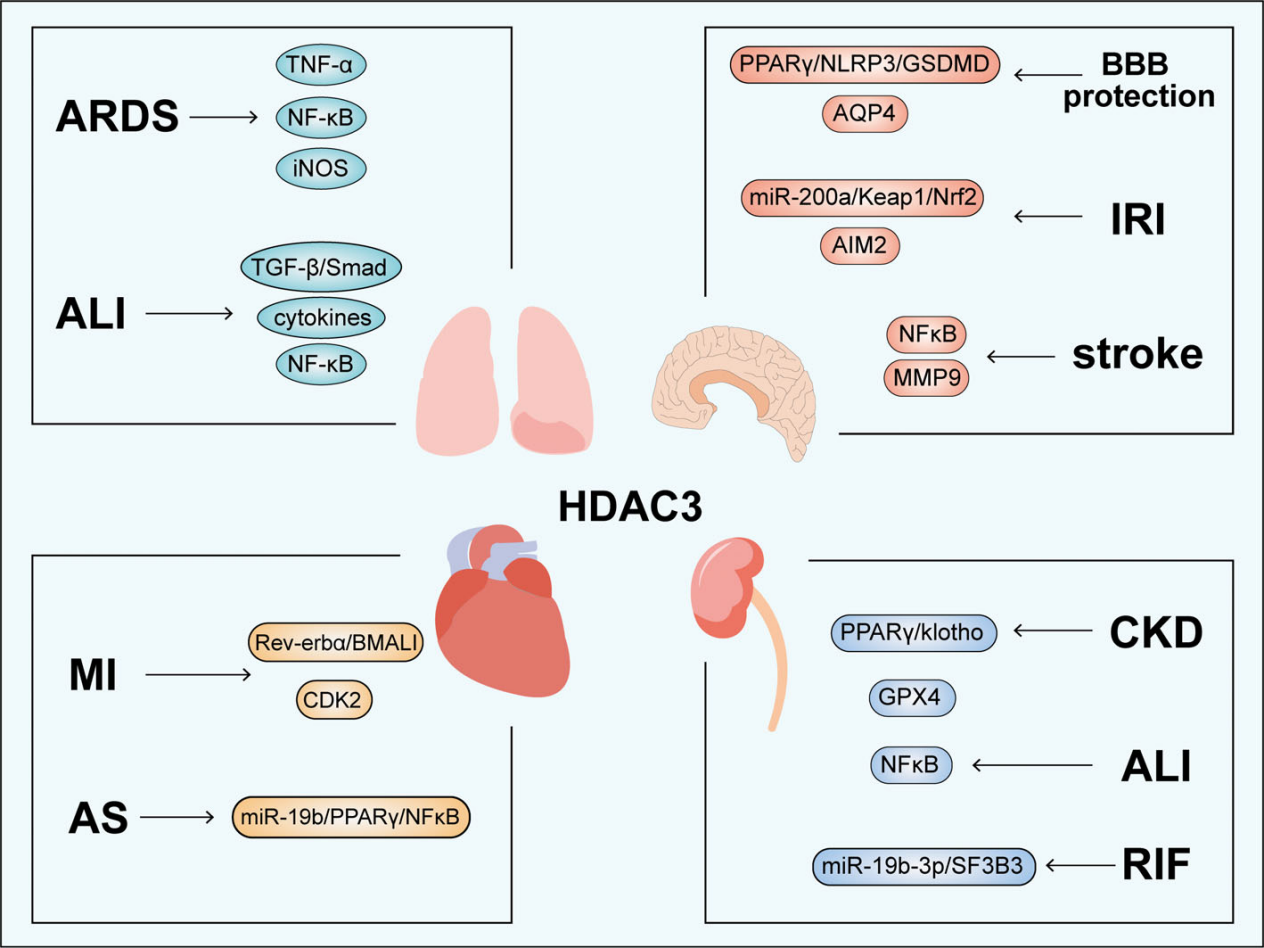
The role of histone deacetylase 3 (HDAC3) in some inflammatory diseases.

As previously mentioned, histone acetylation is intricately associated with the expression of pro‐inflammatory genes.^86^ Due to its regulatory role in inflammatory pathways, HDAC3 exerts a profound impact on the onset and progression of numerous diseases.^38^ Therefore, targeting HDAC3 appears to be crucial for treating multiple inflammatory diseases (Figure 3).

### 4.1 Respiratory diseases

Targeted modulation of inflammation can be implicated in regulating the advancement of inflammation‐associated respiratory diseases. Acute respiratory distress syndrome (ARDS) is induced by sepsis and pneumonia, which is characterized by an exaggerated acute inflammatory response in the pulmonary parenchyma. Nimbolide, a natural terpene lactone, the production of inflammatory cytokines and chemokines through ameliorating NF‐κB and HDAC3 crosstalk regulated by TNF‐α and inhibiting iNOS expression, thereby alleviating the inflammatory symptoms associated with ARDS disease.^87^ Acute lung injury (ALI) is a highly fatal disease mediated by inflammation. HDAC3, as a key determinant in the process of PM2.5‐induced lung injury, mediates the TGF‐β/Smad pathway and subsequently enhances NF‐κB activation, resulting in the release of proinflammatory cytokines and infiltration of inflammatory cells.^70^

Other evidence suggests that the deletion of HDAC3 disrupts the equilibrium of NF‐κB‐dependent nuclear transcription, leading directly to the overexpression of inflammatory cytokines^88^ and exacerbating pulmonary inflammation. LPS‐induced TLR4 activation facilitates the translocation of HDAC3 and NF‐κB to alveolar epithelial nuclei during septic‐induced acute lung injury.^89^ Furthermore, HDAC3 inhibitors have been demonstrated to suppress the expression of various pro‐inflammatory cytokines in macrophages by attenuating NF‐κB activity, thereby effectively mitigating pulmonary inflammation.^87^ Therefore, HDAC3 inhibitors hold significant potential for pre‐therapeutic investigations of various inflammatory lung diseases.

### 4.2 Cardiovascular diseases

Previous studies have identified HDAC3 as a promising novel therapeutic target for cardiovascular diseases. HDAC3 serves as a crucial regulator of circadian rhythm. Deletion of HDAC3 specifically in cardiomyocytes alleviates myocardial infarction in diabetic models by modulating the clock gene Rev‐erbα/BMAL1 pathway.^90^ Treatment with RGFP966 in mice with myocardial infarction promotes miR‐19a‐3p expression while reducing cyclin‐dependent kinase 2 levels within the myocardium, thereby mitigating heart injury.

Atherosclerosis (AS) is widely recognized as the primary cause of morbidity and mortality associated with cardiovascular disease.^91^ HDAC3 is the only upregulated HDAC observed in human atherosclerotic ruptured lesions,^92^ which correlates with inflammatory macrophages. HDAC3 has been identified as a suppressor of AS initiation through its promotion of endothelial cell survival.^93^ In mice, HDAC3 inhibits AS inflammation by suppressing miR‐19b activity, thereby attenuating its binding to PPARγ and degradation of NF‐κB p65.^94^ Various HDACIs can effectively inhibit the progression of AS, which can be attributed to their anti‐inflammatory effects.^95^ However, it is worth noting that certain HDACIs may not always exert positive effects.^14^ Nevertheless, early loss of HDAC3 results in embryonic death and its absence leads to ventricular structural loss and abnormal heart development,^47^ highlighting its non‐enzymatic function's significance as well.

### 4.3 Cerebrovascular and neurodegenerative diseases

Accumulating compelling evidence suggests that HDAC3 plays a pivotal role in regulating neurodegeneration across various disease models. HDAC3 plays a pro‐inflammatory role in cerebrovascular and neurodegenerative diseases, while its inhibition can effectively reduce neuroinflammation through various inflammatory pathways or modulation of the activation and secretion of inflammatory mediators, thereby exerting a neuroprotective effect. The latest evidence demonstrates that the HDAC3 inhibitor BRD3308 modulates microglial pyroptosis and neuroinflammation through the PPARγ/NLRP3/GSDMD axis, thereby ameliorating neurological function following intraventricular haemorrhage in mice.^85^ RGFP966‐mediated preservation of BBB integrity after ischaemia is associated with reduced AQP4 expression and attenuation of neuroinflammation. Selective inhibition of HDAC3 has been shown to improve long‐term functional outcomes after stroke.^96^ HDAC3 has also been shown to promote the progression of neurodegenerative diseases such as Alzheimer's disease,^97^, ^98^ Charcot‐Marie‐Tooth,^99^ Parkinson's disease,^100^ and Huntington's disease.^101^, ^102^ The application of HDACIs can improve related diseases to a certain extent. Notably, the application of HDACIs appears to have both promoting^103^ and inhibitory^104^, ^105^ effects with respect to microglial inflammatory responses, and this difference may be attributed to different functions among the different members of the HDAC family that HDACIs inhibit.

### 4.4 Kidney diseases

HDAC3 is indispensable for renal development and plays a pivotal role in various renal diseases. Renal interstitial fibrosis (RIF) is typically induced by inflammation. The involvement of the HDAC3/miR‐19b‐3p/SF3B3 axis has been substantiated to be crucial in RIF.^106^ The regulation of chronic kidney disease (CKD) by HDAC3 appears to involve the peroxisome PPARγ/Klotho pathway. Klotho is not only an anti‐aging protein and a PPARγ target highly expressed in the kidney but also serves as a biomarker and therapeutic target for CKD.^107^ HDAC3 can interact with transcriptional activators NCoR and NF‐κB, binding to the promoter region of Klotho.^108^ In adenine‐induced CKD mice kidneys, administration of TSA and RGFP966 similarly increased PPARγ acetylation levels while reversing Klotho inhibition, thereby ameliorating CKD.^109^

Inhibition of HDAC3 can exert an anti‐inflammatory effect in various models of kidney disease.^110^, ^111^ The latest evidence suggests that gene knockout of Hdac3 or administration of RGFP966 effectively mitigates fibrosis‐associated renal function loss while decelerating the AKI‐CKD transition.^112^ However, comprehensive research on HDAC3 in renal‐associated inflammatory diseases is lacking, and further studies are needed to enhance the development of targeted therapeutic strategies.

### 4.5 Digestive diseases

HDAC3 has previously been shown to coordinate the maintenance of bacteria‐dependent intestinal homeostasis.^113^, ^114^ Numerous studies have indicated that HDAC3 plays a pivotal role in the pathogenesis of non‐alcoholic steatohepatitis,^115^, ^116^ autoimmune hepatitis,^117^ inflammatory bowel disease (IBD)^118^ and colitis.^119^ Specific deletion of Hdac3 in the gut of mice fed a high‐fat diet has been observed to enhance susceptibility to colitis, highlighting the critical role HDAC3 plays in inflammation‐related digestive diseases.^120^ Recent evidence suggests a positive correlation between increased HDAC3 activity in peripheral blood mononuclear cells and inflammatory markers in type 2 diabetes mellitus patients.^121^ Inhibiting HDAC3 mediated Nrf2 activation in alleviating diabetes‐induced liver injury in mice showed promising results.^66^ The development of selective HDAC3 inhibitors holds great promise for the treatment of inflammatory diseases. Notably, HDACIs have exhibited favourable effects in rodent models of IBD,^122^ colitis,^69^ etc. These findings emphasize the clinical development potential of these drugs.

### 4.6 Other related diseases

HDAC3 has also been shown to play an important regulatory role in other inflammation‐related diseases. The latest evidences show that HDAC3 participates in the progression of osteoarthritis,^123^ rheumatoid arthritis,^34^, ^124^, ^125^ gout inflammation,^126^ ankylosing spondylitis^127^ and other related diseases with its pro‐inflammatory effect, and its key role in inflammation‐related diseases has gradually been paid attention to. It is expected that with further comprehensive research on the pathogenesis of HDAC3 in various inflammation‐related system diseases, selective HDAC3 inhibitors will gradually enter clinical practice. It is anticipated that with further comprehensive research on the pathogenesis of HDAC3 in various inflammation‐associated diseases, selective HDAC3 inhibitors will gradually find their way into clinical practice.

## 5 FUTURE PERSPECTIVE AND CONCLUSION

As a unique member of the HDAC family, shuttling between the nucleus and cytoplasm, HDAC3 plays a pivotal role in integrating diverse environmental signals to regulate cell fate, development, metabolism, and energy homeostasis. These functions are closely associated with its enzymatic and non‐enzymatic activities. This review comprehensively summarizes the crucial involvement of HDAC3 in inflammatory pathways by examining inflammatory mediators and enzymes, inflammatory transcription factors, as well as inflammation‐related diseases.

However, due to the intricate nature of HDAC3 function and limitations in research methodologies, our understanding of the relationship between HDAC3 and inflammatory pathways remains incomplete. Currently, there is limited knowledge regarding the non‐enzymatic mechanisms of HDAC3. Although numerous studies have utilized HDACIs to modulate HDAC3 expression levels for investigating inflammation both in vivo and in vitro, it should be noted that many available HDACIs are pan‐HDACIs. Therefore, careful attention must be paid to both the enzymatic and non‐enzymatic activities of HDAC3. It is challenging to attribute a series of biological effects solely to a single acetylation event within a specific protein. Moreover, when considering the involvement of HDAC3 in regulating inflammation‐associated diseases or states, multiple inflammatory mediators are simultaneously implicated. Although some pro‐inflammatory mediators may be inhibited by HDACIs, they could potentially amplify other inflammatory pathways as well. Our current understanding of acetylation homeostasis and the crucial role played by HDAC3 in inflammation and related diseases strongly suggests that targeting this enzyme for regulating inflammatory mediators and responses holds great promise for treating such conditions effectively. In summary, comprehensive identification of target genes and pathways regulated by HDAC3 is vital for clinical drug development pertaining to inflammatory and metabolic diseases.

## AUTHOR CONTRIBUTIONS

RH, ZH and TZ wrote the main manuscript text, BL provided significant input. MG, NL and QG reviewed and edited the manuscript and approved the final version. All authors have read and approved the manuscript.

## FUNDING INFORMATION

This work was supported by grants from the National Natural Science Foundation of China (Nos. 8210082163 and 82300103), the Fundamental Research Funds for the Central Universities (Nos. 2042021kf0081 and 2042023kf0011) and the Natural Science Foundation of Hubei Province (No. 2020CFA027).

## CONFLICT OF INTEREST STATEMENT

The authors declare that they have no competing interests.

## References

[1] Medzhitov R . The spectrum of inflammatory responses. Science (New York, NY). 2021;374:1070‐1075. doi:10.1126/science.abi5200 34822279

[2] Hotamisligil GS . Inflammation, metaflammation and immunometabolic disorders. Nature. 2017;542:177‐185. doi:10.1038/nature21363 28179656

[3] Meizlish ML , Franklin RA , Zhou X , Medzhitov R . Tissue homeostasis and inflammation. Annu Rev Immunol. 2021;39:557‐581. doi:10.1146/annurev-immunol-061020-053734 33651964

[4] Hunter P . The inflammation theory of disease. The growing realization that chronic inflammation is crucial in many diseases opens new avenues for treatment. EMBO Rep. 2012;13:968‐970. doi:10.1038/embor.2012.142 23044824 PMC3492709

[5] Coussens LM , Werb Z . Inflammation and cancer. Nature. 2002;420:860‐867. doi:10.1038/nature01322 12490959 PMC2803035

[6] Verdin E , Ott M . 50 years of protein acetylation: from gene regulation to epigenetics, metabolism and beyond. Nat Rev Mol Cell Biol. 2015;16:258‐264. doi:10.1038/nrm3931 25549891

[7] Goodson M , Jonas BA , Privalsky MA . Corepressors: custom tailoring and alterations while you wait. Nucl Recept Signal. 2005;3:e003. doi:10.1621/nrs.03003 16604171 PMC1402215

[8] Li J , Wang J , Wang J , et al. Both corepressor proteins SMRT and N‐CoR exist in large protein complexes containing HDAC3. EMBO J. 2000;19:4342‐4350. doi:10.1093/emboj/19.16.4342 10944117 PMC302030

[9] Ning L , Rui X , Bo W , Qing G . The critical roles of histone deacetylase 3 in the pathogenesis of solid organ injury. Cell Death Dis. 2021;12:734. doi:10.1038/s41419-021-04019-6 34301918 PMC8302660

[10] Nguyen HCB , Adlanmerini M , Hauck AK , Lazar MA . Dichotomous engagement of HDAC3 activity governs inflammatory responses. Nature. 2020;584:286‐290. doi:10.1038/s41586-020-2576-2 32760002 PMC7725280

[11] Zhang K , Liu Z , Yao Y , et al. Structure‐based design of a selective class I Histone Deacetylase (HDAC) near‐infrared (NIR) probe for epigenetic regulation detection in triple‐negative breast cancer (TNBC). J Med Chem. 2021;64:4020‐4033. doi:10.1021/acs.jmedchem.0c02161 33745280

[12] Yang X‐J , Seto E . The Rpd3/Hda1 family of lysine deacetylases: from bacteria and yeast to mice and men. Nat Rev Mol Cell Biol. 2008;9:206‐218. doi:10.1038/nrm2346 18292778 PMC2667380

[13] Sauve AA , Wolberger C , Schramm VL , Boeke JD . The biochemistry of sirtuins. Annu Rev Biochem. 2006;75:435‐465. doi:10.1146/annurev.biochem.74.082803.133500 16756498

[14] Yoon S , Eom GH . HDAC and HDAC inhibitor: from cancer to cardiovascular diseases. Chonnam Med J. 2016;52:1‐11. doi:10.4068/cmj.2016.52.1.1 26865995 PMC4742605

[15] Chen X , He Y , Fu W , et al. Histone deacetylases (HDACs) and atherosclerosis: a mechanistic and pharmacological review. Front Cell Dev Biol. 2020;8:581015. doi:10.3389/fcell.2020.581015 33282862 PMC7688915

[16] Emmett MJ , Lazar MA . Integrative regulation of physiology by histone deacetylase 3. Nat Rev Mol Cell Biol. 2019;20:102‐115. doi:10.1038/s41580-018-0076-0 30390028 PMC6347506

[17] Maolanon AR , Madsen AS , Olsen CA . Innovative strategies for selective inhibition of histone deacetylases. Cell Chem Biol. 2016;23:759‐768. doi:10.1016/j.chembiol.2016.06.011 27447046

[18] Adhikari N , Amin SA , Trivedi P , Jha T , Ghosh B . HDAC3 is a potential validated target for cancer: an overview on the benzamide‐based selective HDAC3 inhibitors through comparative SAR/QSAR/QAAR approaches. Eur J Med Chem. 2018;157:1127‐1142. doi:10.1016/j.ejmech.2018.08.081 30179749

[19] Cao F , Zwinderman MRH , Dekker FJ . The process and strategy for developing selective histone deacetylase 3 inhibitors. Molecules (Basel, Switzerland). 2018;23:551. doi:10.3390/molecules23030551 29498635 PMC6017514

[20] Zhang Y , LeRoy G , Seelig HP , Lane WS , Reinberg D . The dermatomyositis‐specific autoantigen Mi2 is a component of a complex containing histone deacetylase and nucleosome remodeling activities. Cell. 1998;95:279‐289. doi:10.1016/s0092-8674(00)81758-4 9790534

[21] Yoon H‐G , Chan DW , Huang Z‐Q , et al. Purification and functional characterization of the human N‐CoR complex: the roles of HDAC3, TBL1 and TBLR1. EMBO J. 2003;22:1336‐1346. doi:10.1093/emboj/cdg120 12628926 PMC151047

[22] Zhang J , Kalkum M , Chait BT , Roeder RG . The N‐CoR‐HDAC3 nuclear receptor corepressor complex inhibits the JNK pathway through the integral subunit GPS2. Mol Cell. 2002;9:611‐623. doi:10.1016/s1097-2765(02)00468-9 11931768

[23] Perissi V , Rosenfeld MG . Controlling nuclear receptors: the circular logic of cofactor cycles. Nat Rev Mol Cell Biol. 2005;6:542‐554. doi:10.1038/nrm1680 15957004

[24] Hu X , Lazar MA . The CoRNR motif controls the recruitment of corepressors by nuclear hormone receptors. Nature. 1999;402:93‐96. doi:10.1038/47069 10573424

[25] Perissi V , Jepsen K , Glass CK , Rosenfeld MG . Deconstructing repression: evolving models of co‐repressor action. Nat Rev Genet. 2010;11:109‐123. doi:10.1038/nrg2736 20084085

[26] Boyer LA , Langer MR , Crowley KA , Tan S , Denu JM , Peterson CL . Essential role for the SANT domain in the functioning of multiple chromatin remodeling enzymes. Mol Cell. 2002;10:935‐942. doi:10.1016/s1097-2765(02)00634-2 12419236

[27] Guenther MG , Yu J , Kao GD , Yen TJ , Lazar MA . Assembly of the SMRT‐histone deacetylase 3 repression complex requires the TCP‐1 ring complex. Genes Dev. 2002;16:3130‐3135. doi:10.1101/gad.1037502 12502735 PMC187500

[28] Sun Z , Feng D , Fang B , et al. Deacetylase‐independent function of HDAC3 in transcription and metabolism requires nuclear receptor corepressor. Mol Cell. 2013;52:769‐782. doi:10.1016/j.molcel.2013.10.022 24268577 PMC3877208

[29] Adrain C , Freeman M . New lives for old: evolution of pseudoenzyme function illustrated by iRhoms. Nat Rev Mol Cell Biol. 2012;13:489‐498. doi:10.1038/nrm3392 22781900

[30] You S‐H , Lim H‐W , Sun Z , Broache M , Won K‐J , Lazar MA . Nuclear receptor co‐repressors are required for the histone‐deacetylase activity of HDAC3 in vivo. Nat Struct Mol Biol. 2013;20:182‐187. doi:10.1038/nsmb.2476 23292142 PMC3565028

[31] Shakespear MR , Halili MA , Irvine KM , Fairlie DP , Sweet MJ . Histone deacetylases as regulators of inflammation and immunity. Trends Immunol. 2011;32:335‐343. doi:10.1016/j.it.2011.04.001 21570914

[32] Chen G , Goeddel DV . TNF‐R1 signaling: a beautiful pathway. Science (New York, NY). 2002;296:1634‐1635. doi:10.1126/science.1071924 12040173

[33] Lundblad LKA , Thompson‐Figueroa J , Leclair T , et al. Tumor necrosis factor‐alpha overexpression in lung disease: a single cause behind a complex phenotype. Am J Respir Crit Care Med. 2005;171:1363‐1370. doi:10.1164/rccm.200410-1349OC 15805183 PMC2718479

[34] Angiolilli C , Kabala PA , Grabiec AM , et al. Histone deacetylase 3 regulates the inflammatory gene expression programme of rheumatoid arthritis fibroblast‐like synoviocytes. Ann Rheum Dis. 2017;76:277‐285. doi:10.1136/annrheumdis-2015-209064 27457515 PMC5264225

[35] Liu H , Wang J , He T , et al. Butyrate: a double‐edged sword for health? Adv Nutri. 2018;9:21‐29. doi:10.1093/advances/nmx009 PMC6333934 29438462

[36] Schioppa T , Nguyen HO , Tiberio L , et al. Inhibition of class I histone deacetylase activity blocks the induction of TNFAIP3 both directly and indirectly via the suppression of endogenous TNF‐α. Int J Mol Sci. 2022;23:9752. doi:10.3390/ijms23179752 36077149 PMC9456523

[37] Cantley MD , Haynes DR . Epigenetic regulation of inflammation: progressing from broad acting histone deacetylase (HDAC) inhibitors to targeting specific HDACs. Inflammopharmacology. 2013;21:301‐307. doi:10.1007/s10787-012-0166-0 23341163

[38] Ryu Y , Kee HJ , Sun S , et al. Class I histone deacetylase inhibitor MS‐275 attenuates vasoconstriction and inflammation in angiotensin II‐induced hypertension. PLoS One. 2019;14:e0213186. doi:10.1371/journal.pone.0213186 30830950 PMC6398866

[39] Wang T‐Y , Chang M‐M , Li Y‐SJ , Huang T‐C , Chien S , Wu C‐C . Maintenance of HDACs and H3K9me3 prevents arterial flow‐induced venous endothelial damage. Front Cell Dev Biol. 2021;9:642150. doi:10.3389/fcell.2021.642150 33898431 PMC8063156

[40] Park D , Kim Y , Kim H , et al. Hyaluronic acid promotes angiogenesis by inducing RHAMM‐TGFβ receptor interaction via CD44‐PKCδ. Mol Cells. 2012;33:563‐574. doi:10.1007/s10059-012-2294-1 22610405 PMC3887750

[41] Sun H‐J , Ren X‐S , Xiong X‐Q , et al. NLRP3 inflammasome activation contributes to VSMC phenotypic transformation and proliferation in hypertension. Cell Death Dis. 2017;8:e3074. doi:10.1038/cddis.2017.470 28981106 PMC5680591

[42] Chen M , Wang H , Chen W , Meng G . Regulation of adaptive immunity by the NLRP3 inflammasome. Int Immunopharmacol. 2011;11:549‐554. doi:10.1016/j.intimp.2010.11.025 21118671

[43] Lu H , Ashiqueali R , Lin CI , et al. Histone deacetylase 3 inhibition decreases cerebral edema and protects the blood‐brain barrier after stroke. Mol Neurobiol. 2023;60:235‐246. doi:10.1007/s12035-022-03083-z 36258136 PMC9758108

[44] Zhang M‐J , Zhao Q‐C , Xia M‐X , et al. The HDAC3 inhibitor RGFP966 ameliorated ischemic brain damage by downregulating the AIM2 inflammasome. FASEB J. 2020;34:648‐662. doi:10.1096/fj.201900394RRR 31914678

[45] Poli G , Fabi C , Bellet MM , et al. Epigenetic mechanisms of inflammasome regulation. Int J Mol Sci. 2020;21:5758. doi:10.3390/ijms21165758 32796686 PMC7460952

[46] Kukreja RC , Kontos HA , Hess ML , Ellis EF . PGH synthase and lipoxygenase generate superoxide in the presence of NADH or NADPH. Circ Res. 1986;59:612‐619. doi:10.1161/01.res.59.6.612 3028671

[47] He R , Liu B , Geng B , Li N , Geng Q . The role of HDAC3 and its inhibitors in regulation of oxidative stress and chronic diseases. Cell Death Discov. 2023;9:131. doi:10.1038/s41420-023-01399-w 37072432 PMC10113195

[48] Peulen O , Gonzalez A , Peixoto P , et al. The anti‐tumor effect of HDAC inhibition in a human pancreas cancer model is significantly improved by the simultaneous inhibition of cyclooxygenase 2. PLoS One. 2013;8:e75102. doi:10.1371/journal.pone.0075102 24040391 PMC3770617

[49] Kwon Y , Kim Y , Eom S , et al. MicroRNA‐26a/−26b‐COX‐2‐MIP‐2 loop regulates allergic inflammation and allergic inflammation‐promoted enhanced tumorigenic and metastatic potential of cancer cells. J Biol Chem. 2015;290:14245‐14266. doi:10.1074/jbc.M115.645580 25907560 PMC4447993

[50] Tong X , Yin L , Giardina C . Butyrate suppresses Cox‐2 activation in colon cancer cells through HDAC inhibition. Biochem Biophys Res Commun. 2004;317:463‐471. doi:10.1016/j.bbrc.2004.03.066 15063780

[51] Jung S‐B , Kim C‐S , Naqvi A , et al. Histone Deacetylase‐3 antagonizes aspirin‐stimulated endothelial nitric oxide production by reversing aspirin‐induced lysine acetylation of endothelial nitric oxide synthase. Circ Res. 2010;107:877‐887. doi:10.1161/CIRCRESAHA.110.222968 20705923 PMC3025612

[52] Zhang M‐X , Zhang C , Shen YH , et al. Effect of 27nt small RNA on endothelial nitric‐oxide synthase expression. Mol Biol Cell. 2008;19:3997‐4005. doi:10.1091/mbc.e07-11-1186 18614799 PMC2526692

[53] Choi W‐S , Seo Y‐B , Shin P‐G , et al. Veratric acid inhibits iNOS expression through the regulation of PI3K activation and histone acetylation in LPS‐stimulated RAW264.7 cells. Int J Mol Med. 2015;35:202‐210. doi:10.3892/ijmm.2014.1982 25352364

[54] de la Vega L , Grishina I , Moreno R , Krüger M , Braun T , Schmitz ML . A redox‐regulated SUMO/acetylation switch of HIPK2 controls the survival threshold to oxidative stress. Mol Cell. 2012;46:472‐483. doi:10.1016/j.molcel.2012.03.003 22503103

[55] Besong EE , Akhigbe TM , Obimma JN , Obembe OO , Akhigbe RE . Acetate abates arsenic‐induced male reproductive toxicity by suppressing HDAC and uric acid‐driven Oxido‐inflammatory NFkB/iNOS/NO response in rats. Biol Trace Elem Res. 2023;202:2672‐2687. doi:10.1007/s12011-023-03860-4 37726447

[56] Cao M , Yang J , Wang X , et al. Sophora subprostrate polysaccharide regulates histone acetylation to inhibit inflammation in PCV2‐infected murine splenic lymphocytes in vitro and in vivo. Int J Biol Macromol. 2021;191:668‐678. doi:10.1016/j.ijbiomac.2021.09.119 34560152

[57] Gidday JM , Gasche YG , Copin J‐C , et al. Leukocyte‐derived matrix metalloproteinase‐9 mediates blood‐brain barrier breakdown and is proinflammatory after transient focal cerebral ischemia. Am J Physiol Heart Circ Physiol. 2005;289:H558‐H568. doi:10.1152/ajpheart.01275.2004 15764676

[58] Culley KL , Hui W , Barter MJ , et al. Class I histone deacetylase inhibition modulates metalloproteinase expression and blocks cytokine‐induced cartilage degradation. Arthritis Rheum. 2013;65:1822‐1830. doi:10.1002/art.37965 23575963

[59] Xiang X , Dong G , Zhu J , Zhang G , Dong Z . Inhibition of HDAC3 protects against kidney cold storage/transplantation injury and allograft dysfunction. Clin Sci. 2022;136:45‐60. doi:10.1042/CS20210823 34918039

[60] Liu J , Li G , Wang X , et al. Droxinostat, a histone deacetylase inhibitor, induces apoptosis in hepatocellular carcinoma cell lines via activation of the mitochondrial pathway and downregulation of FLIP. Transl Oncol. 2016;9:70‐78. doi:10.1016/j.tranon.2016.01.004 26947884 PMC4800063

[61] Dinkova‐Kostova AT , Abramov AY . The emerging role of Nrf2 in mitochondrial function. Free Radic Biol Med. 2015;88:179‐188. doi:10.1016/j.freeradbiomed.2015.04.036 25975984 PMC4726722

[62] Zhang L , Fei M , Wang H , Zhu Y . Sodium aescinate provides neuroprotection in experimental traumatic brain injury via the Nrf2‐ARE pathway. Brain Res Bull. 2020;157:26‐36. doi:10.1016/j.brainresbull.2020.01.019 32014567

[63] Wang B , Zhu X , Kim Y , et al. Histone deacetylase inhibition activates transcription factor Nrf2 and protects against cerebral ischemic damage. Free Radic Biol Med. 2012;52:928‐936. doi:10.1016/j.freeradbiomed.2011.12.006 22226832 PMC6010182

[64] Gu H‐P , Wu X‐F , Gong Y‐T , et al. RGFP966 exerts neuroprotective effect via HDAC3/Nrf2 pathway after surgical brain injury in rats. Heliyon. 2023;9:e18160. doi:10.1016/j.heliyon.2023.e18160 37539293 PMC10395478

[65] Zhao Q , Zhang F , Yu Z , et al. HDAC3 inhibition prevents blood‐brain barrier permeability through Nrf2 activation in type 2 diabetes male mice. J Neuroinflammation. 2019;16:103. doi:10.1186/s12974-019-1495-3 31101061 PMC6525453

[66] Zhang J , Xu Z , Gu J , et al. HDAC3 inhibition in diabetic mice may activate Nrf2 preventing diabetes‐induced liver damage and FGF21 synthesis and secretion leading to aortic protection. Am J Physiol Endocrinol Metab. 2018;315:E150‐E162. doi:10.1152/ajpendo.00465.2017 29634312

[67] Rajendran P , Dashwood W‐M , Li L , et al. Nrf2 status affects tumor growth, HDAC3 gene promoter associations, and the response to sulforaphane in the colon. Clin Epigenetics. 2015;7:102. doi:10.1186/s13148-015-0132-y 26388957 PMC4575421

[68] Sun W , Zhang N , Liu B , et al. HDAC3 inhibitor RGFP966 ameliorated neuroinflammation in the Cuprizone‐induced demyelinating mouse model and LPS‐stimulated BV2 cells by downregulating the P2X7R/STAT3/NF‐κB65/NLRP3 activation. ACS Chem Nerosci. 2022;13:2579‐2598. doi:10.1021/acschemneuro.1c00826 35947794

[69] Falkenberg KJ , Johnstone RW . Histone deacetylases and their inhibitors in cancer, neurological diseases and immune disorders. Nat Rev Drug Discov. 2014;13:673‐691. doi:10.1038/nrd4360 25131830

[70] Gu L‐Z , Sun H , Chen J‐H . Histone deacetylases 3 deletion restrains PM2.5‐induced mice lung injury by regulating NF‐κB and TGF‐β/Smad2/3 signaling pathways. Biomed Pharmacother. 2017;85:756‐762. doi:10.1016/j.biopha.2016.11.094 27919737

[71] Ghizzoni M , Haisma HJ , Maarsingh H , Dekker FJ . Histone acetyltransferases are crucial regulators in NF‐κB mediated inflammation. Drug Discov Today. 2011;16:504‐511. doi:10.1016/j.drudis.2011.03.009 21477662 PMC5218544

[72] Ziesché E , Kettner‐Buhrow D , Weber A , et al. The coactivator role of histone deacetylase 3 in IL‐1‐signaling involves deacetylation of p65 NF‐κB. Nucleic Acids Res. 2013;41:90‐109. doi:10.1093/nar/gks916 23087373 PMC3592411

[73] Leus NGJ , Zwinderman MRH , Dekker FJ . Histone deacetylase 3 (HDAC 3) as emerging drug target in NF‐κB‐mediated inflammation. Curr Opin Chem Biol. 2016;33:160‐168. doi:10.1016/j.cbpa.2016.06.019 27371876 PMC5019345

[74] Tang J , Cho NW , Cui G , et al. Acetylation limits 53BP1 association with damaged chromatin to promote homologous recombination. Nat Struct Mol Biol. 2013;20:317‐325. doi:10.1038/nsmb.2499 23377543 PMC3594358

[75] Yang T , Zhang Y , Chen L , et al. The potential roles of ATF family in the treatment of Alzheimer's disease. Biomed Pharmacother. 2023;161:114544. doi:10.1016/j.biopha.2023.114544 36934558

[76] Chen M , Liu Y , Yang Y , et al. Emerging roles of activating transcription factor (ATF) family members in tumourigenesis and immunity: implications in cancer immunotherapy. Genes Diseases. 2022;9:981‐999. doi:10.1016/j.gendis.2021.04.008 35685455 PMC9170601

[77] Steer JH , Kroeger KM , Abraham LJ , Joyce DA . Glucocorticoids suppress tumor necrosis factor‐alpha expression by human monocytic THP‐1 cells by suppressing transactivation through adjacent NF‐kappa B and c‐Jun‐activating transcription factor‐2 binding sites in the promoter. J Biol Chem. 2000;275:18432‐18440. doi:10.1074/jbc.M906304199 10748079

[78] Saccani S , Natoli G . Dynamic changes in histone H3 Lys 9 methylation occurring at tightly regulated inducible inflammatory genes. Genes Dev. 2002;16:2219‐2224. doi:10.1101/gad.232502 12208844 PMC186673

[79] Mahlknecht U , Will J , Varin A , Hoelzer D , Herbein G . Histone deacetylase 3, a class I histone deacetylase, suppresses MAPK11‐mediated activating transcription factor‐2 activation and represses TNF gene expression. J Immunol. 2004;173:3979‐3990. doi:10.4049/jimmunol.173.6.3979 15356147

[80] Yousefnia S , Momenzadeh S , Seyed Forootan F , Ghaedi K , Nasr Esfahani MH . The influence of peroxisome proliferator‐activated receptor γ (PPARγ) ligands on cancer cell tumorigenicity. Gene. 2018;649:14‐22. doi:10.1016/j.gene.2018.01.018 29369787

[81] Gao Q , Wei A , Chen F , et al. Enhancing PPARγ by HDAC inhibition reduces foam cell formation and atherosclerosis in ApoE deficient mice. Pharmacol Res. 2020;160:105059. doi:10.1016/j.phrs.2020.105059 32621955

[82] Yao Y , Liu Q , Adrianto I , et al. Histone deacetylase 3 controls lung alveolar macrophage development and homeostasis. Nat Commun. 2020;11:3822. doi:10.1038/s41467-020-17630-6 32732898 PMC7393351

[83] Jiang X , Ye X , Guo W , Lu H , Gao Z . Inhibition of HDAC3 promotes ligand‐independent PPARγactivation by protein acetylation. J Mol Endocrinol. 2014;53:191‐200. doi:10.1530/JME-14-0066 24982244 PMC4391273

[84] Liu X , Jiang C , Liu G , et al. Sodium butyrate protects against oxidative stress in human nucleus pulposus cells via elevating PPARγ‐regulated Klotho expression. Int Immunopharmacol. 2020;85:85. doi:10.1016/j.intimp.2020.106657 32554208

[85] Li Y , Liu C , Wang G , et al. HDAC3 inhibitor (BRD3308) modulates microglial pyroptosis and neuroinflammation through PPARγ/NLRP3/GSDMD to improve neurological function after intraventricular hemorrhage in mice. Neuropharmacology. 2023;237:109633. doi:10.1016/j.neuropharm.2023.109633 37327970

[86] Daskalaki MG , Tsatsanis C , Kampranis SC . Histone methylation and acetylation in macrophages as a mechanism for regulation of inflammatory responses. J Cell Physiol. 2018;233:6495‐6507. doi:10.1002/jcp.26497 29574768 PMC13482559

[87] Pooladanda V , Thatikonda S , Bale S , et al. Nimbolide protects against endotoxin‐induced acute respiratory distress syndrome by inhibiting TNF‐α mediated NF‐κB and HDAC‐3 nuclear translocation. Cell Death Dis. 2019;10:81. doi:10.1038/s41419-018-1247-9 30692512 PMC6349848

[88] Mullican SE , Gaddis CA , Alenghat T , et al. Histone deacetylase 3 is an epigenomic brake in macrophage alternative activation. Genes Dev. 2011;25:2480‐2488. doi:10.1101/gad.175950.111 22156208 PMC3243058

[89] Akira S , Takeda K . Toll‐like receptor signalling. Nat Rev Immunol. 2004;4:499‐511. doi:10.1038/nri1391 15229469

[90] Griffett K , Hayes ME , Boeckman MP , Burris TP . The role of REV‐ERB in NASH. Acta Pharmacol Sin. 2022;43:1133‐1140. doi:10.1038/s41401-022-00883-w 35217816 PMC9061770

[91] Wei X , Ying M , Dehaini D , et al. Nanoparticle functionalization with platelet membrane enables multifactored biological targeting and detection of atherosclerosis. ACS Nano. 2018;12:109‐116. doi:10.1021/acsnano.7b07720 29216423 PMC5859122

[92] Hoeksema MA , Gijbels MJ , Van den Bossche J , et al. Targeting macrophage histone deacetylase 3 stabilizes atherosclerotic lesions. EMBO Mol Med. 2014;6:1124‐1132. doi:10.15252/emmm.201404170 25007801 PMC4197860

[93] Zampetaki A , Zeng L , Margariti A , et al. Histone deacetylase 3 is critical in endothelial survival and atherosclerosis development in response to disturbed flow. Circulation. 2010;121:132‐142. doi:10.1161/CIRCULATIONAHA.109.890491 20026773

[94] Wang J , Xu X , Li P , Zhang B , Zhang J . HDAC3 protects against atherosclerosis through inhibition of inflammation via the microRNA‐19b/PPARγ/NF‐κB axis. Atherosclerosis. 2021;323:1‐12. doi:10.1016/j.atherosclerosis.2021.02.013 33756273

[95] Kulthinee S , Yano N , Zhuang S , Wang L , Zhao TC . Critical functions of histone deacetylases (HDACs) in modulating inflammation associated with cardiovascular diseases. Pathophysiology. 2022;29:471‐485. doi:10.3390/pathophysiology29030038 35997393 PMC9397025

[96] Matheson R , Chida K , Lu H , et al. Neuroprotective effects of selective inhibition of histone deacetylase 3 in experimental stroke. Transl Stroke Res. 2020;11:1052‐1063. doi:10.1007/s12975-020-00783-3 32016769

[97] Davis N , Taylor B , Abelleira‐Hervas L , et al. Histone deacetylase‐3 regulates the expression of the amyloid precursor protein and its inhibition promotes neuroregenerative pathways in Alzheimer's disease models. FASEB J. 2024;38:e23659. doi:10.1096/fj.202301762RR 38733301

[98] Marinho D , Ferreira IL , Lorenzoni R , Cardoso SM , Santana I , Rego AC . Reduction of class I histone deacetylases ameliorates ER‐mitochondria cross‐talk in Alzheimer's disease. Aging Cell. 2023;22:e13895. doi:10.1111/acel.13895 37358017 PMC10410063

[99] Prior R , Verschoren S , Vints K , et al. HDAC3 inhibition stimulates myelination in a CMT1A mouse model. Mol Neurobiol. 2022;59:3414‐3430. doi:10.1007/s12035-022-02782-x 35320455 PMC9148289

[100] Kumar V , Kundu S , Singh A , Singh S . Understanding the role of histone deacetylase and their inhibitors in neurodegenerative disorders: current targets and future perspective. Curr Neuropharmacol. 2022;20:158‐178. doi:10.2174/1570159X19666210609160017 34151764 PMC9199543

[101] Hecklau K , Mueller S , Koch SP , et al. The effects of selective inhibition of histone deacetylase 1 and 3 in Huntington's disease mice. Front Mol Neurosci. 2021;14:616886. doi:10.3389/fnmol.2021.616886 33679321 PMC7925995

[102] Suelves N , Kirkham‐McCarthy L , Lahue RS , Ginés S . A selective inhibitor of histone deacetylase 3 prevents cognitive deficits and suppresses striatal CAG repeat expansions in Huntington's disease mice. Sci Rep. 2017;7:6082. doi:10.1038/s41598-017-05125-2 28729730 PMC5519595

[103] Meleady L , Towriss M , Kim J , et al. Histone deacetylase 3 regulates microglial function through histone deacetylation. Ciba F Symp. n.d., 2023;18:2241008. doi:10.1080/15592294.2023.2241008 PMC10392760 37506371

[104] Durham BS , Grigg R , Wood IC . Inhibition of histone deacetylase 1 or 2 reduces induced cytokine expression in microglia through a protein synthesis independent mechanism. J Neurochem. 2017;143:214‐224. doi:10.1111/jnc.14144 28796285

[105] Wang C , Shen D , Hu Y , et al. Selective targeting of class I HDAC reduces microglial inflammation in the entorhinal cortex of young APP/PS1 mice. Int J Mol Sci. 2023;24:4805. doi:10.3390/ijms24054805 36902234 PMC10003411

[106] Hu L , Yang K , Mai X , Wei J , Ma C . Depleted HDAC3 attenuates hyperuricemia‐induced renal interstitial fibrosis via miR‐19b‐3p/SF3B3 axis. Cell Cycle. 2022;21:450‐461. doi:10.1080/15384101.2021.1989899 35025700 PMC8942505

[107] Ja N , Mc H . Potential application of klotho in human chronic kidney disease. Bone. 2017;100:41‐48. doi:10.1016/j.bone.2017.01.017 28115282 PMC5474175

[108] Corrales P , Izquierdo‐Lahuerta A , Medina‐Gómez G . Maintenance of kidney metabolic homeostasis by PPAR gamma. Int J Mol Sci. 2018;19:2063. doi:10.3390/ijms19072063 30012954 PMC6073436

[109] Lin W , Zhang Q , Liu L , Yin S , Liu Z , Cao W . Klotho restoration via acetylation of peroxisome proliferation‐activated receptor γ reduces the progression of chronic kidney disease. Kidney Int. 2017;92:669‐679. doi:10.1016/j.kint.2017.02.023 28416226

[110] Liu L , Lin W , Zhang Q , Cao W , Liu Z . TGF‐β induces miR‐30d down‐regulation and podocyte injury through Smad2/3 and HDAC3‐associated transcriptional repression. J Mol Med (Berl). 2016;94:291‐300. doi:10.1007/s00109-015-1340-9 26432290

[111] Li Q , Ge C , Tan J , et al. Juglanin protects against high fat diet‐induced renal injury by suppressing inflammation and dyslipidemia via regulating NF‐κB/HDAC3 signaling. Int Immunopharmacol. 2021;95:107340. doi:10.1016/j.intimp.2020.107340 33667999

[112] Zhang L , Chen F , Dong J , et al. HDAC3 aberration‐incurred GPX4 suppression drives renal ferroptosis and AKI‐CKD progression. Redox Biol. 2023;68:102939. doi:10.1016/j.redox.2023.102939 37890360 PMC10638610

[113] Alenghat T , Osborne LC , Saenz SA , et al. Histone deacetylase 3 coordinates commensal‐bacteria‐dependent intestinal homeostasis. Nature. 2013;504:153‐157. doi:10.1038/nature12687 24185009 PMC3949438

[114] Oh SK , Kim D , Kim K , et al. RORα is crucial for attenuated inflammatory response to maintain intestinal homeostasis. Proc Natl Acad Sci U S A. 2019;116:21140‐21149. doi:10.1073/pnas.1907595116 31570593 PMC6800319

[115] Sun Z , Miller RA , Patel RT , et al. Hepatic Hdac3 promotes gluconeogenesis by repressing lipid synthesis and sequestration. Nat Med. 2012;18:934‐942. doi:10.1038/nm.2744 22561686 PMC3411870

[116] Papazyan R , Sun Z , Kim YH , et al. Physiological suppression of Lipotoxic liver damage by complementary actions of HDAC3 and SCAP/SREBP. Cell Metab. 2016;24:863‐874. doi:10.1016/j.cmet.2016.10.012 27866836 PMC5159233

[117] Xia D , Chen D , Cai T , et al. Nimbolide attenuated the inflammation in the liver of autoimmune hepatitis's mice through regulation of HDAC3. Toxicol Appl Pharmacol. 2022;434:115795. doi:10.1016/j.taap.2021.115795 34780724

[118] Che N , Zhang Y , Zhang S , et al. Macrophagic HDAC3 inhibition ameliorates dextran sulfate sodium induced inflammatory bowel disease through GBP5‐NLRP3 pathway. Int J Med Sci. 2024;21:1385‐1398. doi:10.7150/ijms.94592 38903915 PMC11186415

[119] Zhang F , Qi L , Feng Q , et al. HIPK2 phosphorylates HDAC3 for NF‐κB acetylation to ameliorate colitis‐associated colorectal carcinoma and sepsis. Proc Natl Acad Sci U S A. 2021;118:e2021798118. doi:10.1073/pnas.2021798118 34244427 PMC8285910

[120] Ng I , Luk IY , Nightingale R , et al. Intestinal‐specific Hdac3 deletion increases susceptibility to colitis and small intestinal tumor development in mice fed a high‐fat diet. Am J Physiol Gastrointest Liver Physiol. 2023;325:G508‐G517. doi:10.1152/ajpgi.00160.2023 37788331

[121] Sathishkumar C , Prabu P , Balakumar M , et al. Augmentation of histone deacetylase 3 (HDAC3) epigenetic signature at the interface of proinflammation and insulin resistance in patients with type 2 diabetes. Clin Epigenetics. 2016;8:125. doi:10.1186/s13148-016-0293-3 27904654 PMC5122206

[122] Felice C , Lewis A , Armuzzi A , Lindsay JO , Silver A . Review article: selective histone deacetylase isoforms as potential therapeutic targets in inflammatory bowel diseases. Aliment Pharmacol Ther. 2015;41:26‐38. doi:10.1111/apt.13008 25367825

[123] Fu B , Shen J , Zou X , et al. Matrix stiffening promotes chondrocyte senescence and the osteoarthritis development through downregulating HDAC3. Bone Res. 2024;12:32. doi:10.1038/s41413-024-00333-9 38789434 PMC11126418

[124] Chen H , Fu X , Wu X , et al. Gut microbial metabolite targets HDAC3‐FOXK1‐interferon axis in fibroblast‐like synoviocytes to ameliorate rheumatoid arthritis. Bone Res. 2024;12:31. doi:10.1038/s41413-024-00336-6 38782893 PMC11116389

[125] Yao F , Zhao Y , Yu Q , et al. Extracellular CIRP induces abnormal activation of fibroblast‐like synoviocytes from patients with RA via the TLR4‐mediated HDAC3 pathways. Int Immunopharmacol. 2024;128:111525. doi:10.1016/j.intimp.2024.111525 38218010

[126] Yang Q‐B , Zhang M‐Y , Yang L , Wang J , Mi Q‐S , Zhou J‐G . Deficiency of histone deacetylases 3 in macrophage alleviates monosodium urate crystals‐induced gouty inflammation in mice. Arthritis Res Ther. 2024;26:96. doi:10.1186/s13075-024-03335-4 38711064 PMC11071232

[127] Jiang Y , Wang L . Role of histone deacetylase 3 in ankylosing spondylitis via negative feedback loop with microRNA‐130a and enhancement of tumor necrosis factor‐1α expression in peripheral blood mononuclear cells. Mol Med Rep. 2016;13:35‐40. doi:10.3892/mmr.2015.4494 26531724 PMC4686114

